# How do people with physical disabilities want to construct virtual identities with avatars?

**DOI:** 10.3389/fpsyg.2022.994786

**Published:** 2022-09-13

**Authors:** Jaeyoung Park, Seongcheol Kim

**Affiliations:** School of Media and Communication, Korea University, Seoul, South Korea

**Keywords:** avatar, virtual identity, people with disability, social representations theory, core-periphery analysis

## Abstract

In the virtual world, people can reconstruct their identity the way they want with avatars. Many expect the high degree of freedom in avatar customization will give new chances to socially marginalized people experiencing discrimination against their physical traits. Accordingly, research on a virtual embodiment of marginalized people has been steady with increased interest in equity and inclusion. However, even discourse alienates people with disabilities. In addition, there are few studies on the virtual representations of people with disabilities. Therefore, this paper explores the shared perception of avatar-based disability representations among people with disability to help understand how they want to construct their disability with avatars. The study also gives direction for a barrier-free virtual world. We conducted semi-structured in-depth interviews with people with physical impairments who used virtual world services and performed a core-periphery analysis of social representations. This study expands the range of academic adoption of the social representations theory and provides insights for stakeholders such as service providers to design an inclusive virtual world.

## Introduction

“On the Internet, nobody knows you at all; on the Internet, nobody knows what your race is or your gender. That whole color and gender-blindness is a positive aspect for a lot of people. They feel welcome. Certainly, this goes for people with disabilities.” (Gilmore,1996, as cited in Jordan, 2000, p. 66)

In the virtual world, people can illustrate themselves in the way they want. As a result, the real-world identity, often largely affected by physical attributes, could be absent online. At the heart of such embodiment lies an avatar, a digital and graphical agency of users in the virtual world (Bente et al., 2008). There is no consensus on how people construct their virtual identities with avatars. Some reflect characteristics of the actual self (Nowak and Rauh, 2005), while others make ideal selves with avatars (Messinger et al., 2008). People without physical constraints would not have critical difficulties constructing avatars that reflect their real-world appearance. However, those constrained by their physical traits would have some difficulties.

Similar to two opposite aspects of virtual identity establishment, socially marginalized people decide whether to mirror their physical selves. Their choices are distinct from people without constraints since they have experienced discrimination as people of color, women, or people with disabilities. As they have faced barriers because of physical attributes, marginalized people may be more concerned about displaying or abandoning their physical traits with avatars. Accordingly, research on the avatar-based representation of marginalized people has been steady with increased interest in equity and inclusion focusing on female and colored populations (Cassell and Jenkins, 2000; Kafai et al., 2010; Lee and Park, 2011; Martey and Consalvo, 2011; Todd, 2012; Lee, 2014). However, even discourse alienates people with disabilities (PWD). In addition, there are few studies on the virtual representations of PWD. Therefore, this study focused on PWD and their virtual embodiment of disability to fill this research gap.

Few studies deal with avatar-based disability representation of PWD. Some studies on avatar-based disability representations focused on the role of the avatar in a specific context, such as job searching (Davis and Chansiri, 2018), or emphasized the significance of technical support for avatar creation (Davis and Stanovsek, 2021) with collected data from a single virtual world service, Second Life. According to Alsop (2022), the global virtual world market was worth 38.85 billion USD in 2021 and seems to soar to 678.8 billion USD by 2030. As the virtual world becomes everyday life, further research on avatar-based disability representation of PWD in more general context would be necessary. This paper emphasizes the significance of avatars in the virtual world and investigates shared opinions of PWD on how they want to construct their identity in the virtual world with avatars based on social representations theory (SRT). The SRT explains that shared social representations between a group have a decisive influence on whether members of a group accept the social object (Moscovici, 1984). Therefore, this study gives direction for an inclusive virtual world for PWD by identifying their opinions on disability representations with avatars, the starting point of the virtual world.

This paper begins with an overview of disability embodiment with avatars in academia and industry, followed by a description of the study’s methodological framework, social representations theory. Next, the research method section explains the process of in-depth interviews for data collection and analyses of data, including content and core-periphery analyses to identify the perception structure of PWD on disability embodiment in the virtual world. Next, we present the analysis results in the form of a map. Then we interpret the map in detail in the results section. Finally, we discuss the theoretical and practical implications highlighted by examining the social representations map on avatar-based disability embodiment with suggestions for future studies.

## Theoretical background

### Literature review on avatar-based disability representations

With the development of technology, social interactions on the Internet have become universal on a text-based basis, and the role of avatars as digital interactants has become important (Bente et al., 2008). Since the avatar represents oneself in an online condition, the avatar-creation process is in the course of the identity selection and expression process (Sung et al., 2011). As technology advances allowing character customization, the level of self-expression through avatars has increased. In customization conditions, individuals can select resources for avatar illustration to achieve their strategic goal of self-expression (Sundar and Marathe, 2010). Therefore, users take chances to decide on every physical attribute of their virtual body, avatars.

The question is whether this barrier-free characteristic of avatar creation will give a new chance to socially marginalized groups. Researchers have continuously conducted studies on the avatar-based underrepresentations of gender (Cassell and Jenkins, 2000; Todd, 2012) or racial minorities (Kafai et al., 2010; Lee and Park, 2011; Martey and Consalvo, 2011; Lee, 2014) in the virtual world. Some researchers pointed out the problems of social minorities’ underrepresentations. For example, Cassell and Jenkins (2000) criticized the dominance of male characters leading females away from enjoying the virtual world service game. Similarly, non-white people were less willing to disclose the color of their physical skin with avatars when they were in a situation with low racial diversity, unlike the white who were free from the skin color of other avatars (Lee and Park, 2011; Lee, 2014). Lee and Park (2011) explained that non-white people might perceive the white-dominant virtual world as an identity threat and keep their distance.

So, to allow socially marginalized people to use the virtual world services, the efforts must precede the services to make them visible within the service. However, insufficient prior studies identified implicit reasons for minorities’ abandonment of physical traits and how they prefer to display their characteristics. For example, Martey et al. (2014) investigated the reasons for choosing gender-switched avatars in an online game. They argued that players switch their gender for a strategic reason rather than reasons related to identity expression or gender stigma. However, it is hard to generalize this result to current virtual world services, which support the high level of autonomy in using rather than providing specific missions for users to solve like online games. Furthermore, Lee (2014) found that improving the level of avatar-based diversity representations in the virtual world enhances black participants’ willingness to reveal their race with their avatars. However, it is not certain that we would find similar relationships in the context of disability representations.

Academia has paid little attention to the avatar-based representations of disability. In one study, Forman et al. (2011) empirically examined the users with disability for Second Life, the initial virtual world platform, to explore the embodiment of disability and gender identity in a virtual world environment. They found that the virtual world still marginalized disability, and disabled users joined with others who disclosed a disability on their avatars. However, the study’s limitation was that it could not present an interpretation of the phenomenon since it was exploratory. In another study, Davis and Stanovsek (2021) revealed a complex effect of virtual world technologies on the inclusion of the PWD in the virtual environment, but they focused more on technology.

Davis and Chansiri (2018) also explored the influence of the avatar of PWD in a specific context, job searching, and online social interactions. They categorized PWD’s avatar-based identity representations into three paradigms: (1) reconstruction of virtual identity, (2) combination of virtual and physical identity, and (3) employment of physical identity. The first paradigm means people abandoned their physical identity in avatar-based virtual identity construction. The second paradigm, a combination of virtual and physical identity, indicates that people selectively reveal their physical traits instead of embodying every aspect of the actual self. The third paradigm involves people who try to embody all aspects of themselves with avatars. Applying the three paradigms to the context of PWD, the first paradigm accorded with the abandonment of disability, while the third paradigm corresponded with the display of disability. The second paradigm indicates a selective expression of physical flaws depending on the situation. For example, Davis and Stanovsek (2021) introduced a case in which a user with a mental illness put a brain sling on her avatar to avoid risks so others would consider her in communication. Even though a former case introduced the user with an invisible mental disability, it is necessary to explore whether we can apply the second paradigm to PWD. In this study, to fill the research gap, we tried to investigate the perceptions of PWD of how they want to construct their identity in the general virtual world with avatars.

### Avatar-based disability representations in the virtual world services

Second Life is a virtual world mirroring the real world and the most selected virtual world service for previous studies on avatar-based minority identity representations (Forman et al., 2011; Stendal et al., 2011; Davis and Stanovsek, 2021). A virtual world service resembling the physical world is different from games which are popular virtual world services focused on defeating monsters or upgrading characters. In this service, individuals represented as avatars interact with others and explore the space resembling the real world with extreme freedom (Forman et al., 2011). Especially people with disabilities constrained by physical traits in the real world can enjoy the desirable life distinct from actual life. They can attend all activities they want and associate with different people at different levels in this world (Stendal et al., 2011).

Based on these distinctive characteristics, Second Life received significant attention from researchers. When the virtual world services recreating the real world were not yet common, they preemptively commercialized Second Life in 2003. Therefore, most studies on the avatar-based representations of PWD were against the backdrop of Second Life (Forman et al., 2011; Davis and Chansiri, 2018; Davis and Stanovsek, 2021). However, using only the context of Second Life is a limitation of the studies since its platform characteristics can limit the results. Recently, several companies are scrambling to introduce virtual world services such as Gather.town or ZEPETO reproducing the real world. Hence, it can be the appropriate time to explore the perception of PWD on avatars on a more general level rather than focusing on users of a specific service.

As technology advanced, technological affordances for avatar creation allow people to express more diverse characteristics with given identifiers. Accordingly, virtual world platforms satisfy consumers’ needs for self-expression by providing various skin colors, hair colors, fashion items, etc. This study examined identifiers of ZEPETO, Gather.town, and ifland, three popular virtual world services currently being serviced in Korea. ZEPETO and ifland provide 3D avatars, so the identifiers are more sophisticated and diverse than Gather.town, which uses 2D avatars.

**Figure 1:**
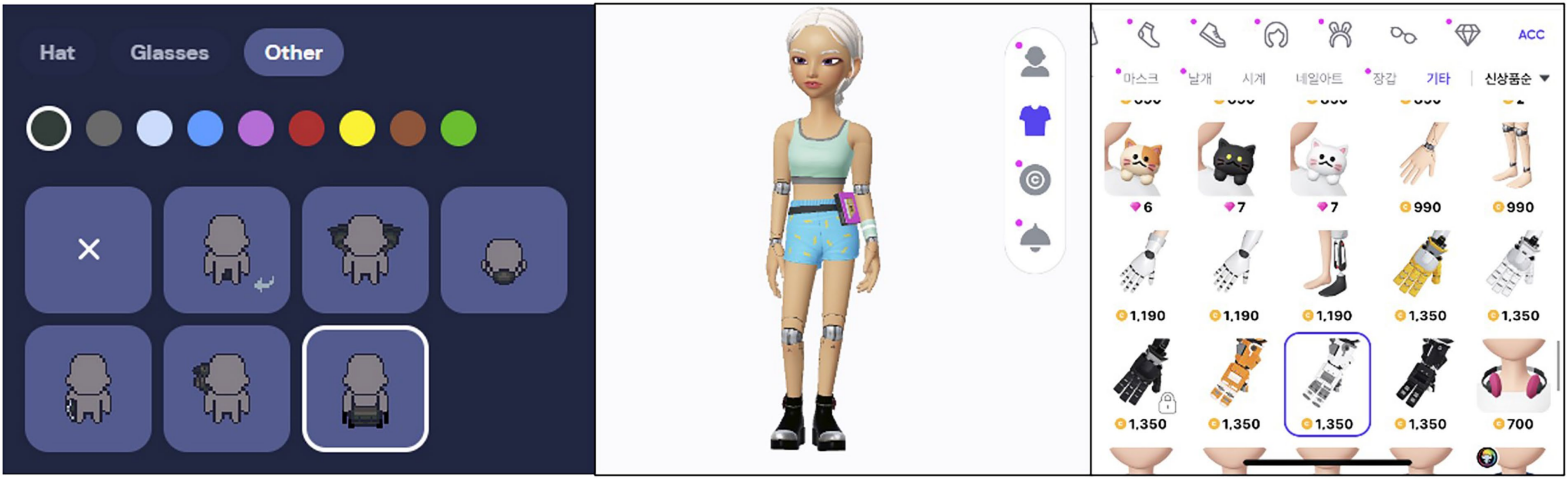
Identifiers for physical disability in Gather.town (left) and ZEPETO. Left Image: Reproduced with permission from Gather Presence, Inc., available at https://www.gather.town/. Right Image: Reproduced with permission from NAVER Z Corp., available at https://zepeto.me/.

The services provide identifiers for displaying physical disability differently. Gather.town and ZEPETO provide physical constraint-related identifiers, while ifland does not offer any option. There is also a difference in describing physical flaws. Gather.town describes disability in a relatively realistic way: identifiers of prosthetic arms, wheelchairs, and cochlear implants resemble each aid’s physical shape. The number of identifiers is small due to the nature of simple forms of 2D avatars. In the case of ZEPETO, unlike Gather.town, it provides forms of artificial arms and legs shaped like cyborgs instead of the typical form. ZEPETO selects fantasy descriptions for illustrating disability, so disability aids such as wheelchairs do not exist in options. Figure 1 shows the physical disability identifiers in Gather.town and ZEPETO.

### Social representations theory

We adopted the social representations theory to explore PWD’s understanding of avatar-based disability representations in virtual world platforms. SRT indicates that one reconstructs a shared understanding of a social object among members with similarities in social and historical contexts (Doise et al., 1993). The theory explains that social representations of objects guide people’s social practices because each individual is a community member at their root (Moscovici, 1984).

This paper employed SRT for three reasons. First, the theory is appropriate for investigating the collective understanding of a specific social group on a social object. PWD as a social minority group may have common ideas about virtual avatars and representations of their physical traits. Exploring the shared understanding from the group is important because it leads to real practice with an object (Augoustinos and Walker, 1995). Therefore, SRT can be a proper theoretical framework for exploring PWD’s collective perception of avatar-based disability representations.

Second, previous studies have successfully applied SRT to investigate how community members perceive a novel social object within a structured shared understanding. For example, Jang et al. (2021) investigated social representations of chatbot services in the financial industry, which is in the initial adoption stage. Moreover, Jung and Pawlowski (2014) adopted SRT to identify users’ understanding of virtual consumption, a novel social activity. Avatar-based disability representations in the virtual world is an emerging media that opens a new world to people with restriction in the real world. PWD are also unfamiliar with showing themselves in avatar forms. Therefore, SRT, which explains shared thoughts of a novel or unfamiliar social object, seems to be a proper approach. It incorporates common opinions of PWD on avatar-based disability representations available through technical development.

Third, exploratory studies have widely selected SRT. SRT is an effective theory for tracing multidimensions of collective thoughts on a social object (Abric, 2001). Since avatar-based disability representations in the virtual world are in the early stage, an exploratory approach with SRT is more appropriate than a confirmatory design.

The SRT consists of a central core and peripheral elements (Abric, 1993), including attributes, perceptions, feelings, and attitudes toward social objects. While core elements generate meaning and value for other elements, one organizes and adaptively interprets peripheral with core elements (Moliner, 1995; Abric, 2001). Researchers have widely employed core-periphery analysis which provides systematic explanations of conceptual components of representations in social representations theory studies (Ahn and Jung, 2016; Jang et al., 2021).

Given this background, we decided to explore how PWD perceive their avatar-based disability representations for designing successful disability identifiers and a barrier-free virtual world. In particular, we can refer to the accurate understanding of PWD disability embodiment in the virtual world to find the future direction of a virtual world that can encompass marginalized and undervalued groups in the offline world. Accordingly, this paper came up with the following research questions:

RQ: How do people with physical disabilities want to construct virtual identities with avatars?

RQ_1_: Do people with disabilities want to disclose their physical flaws with avatars in the virtual world?

RQ_2_: Why do people with disabilities want or not want to describe their physical flaws with avatars in the virtual world?

RQ_3_: What is the preferred way for people with disabilities to describe their physical flaws with avatars in the virtual world?

## Research method

To understand PWD’s opinions on avatar-based representations of physical flaws in a virtual world, we employed a core-periphery analysis of SRT. We interviewed PWD with experience using an avatar-based virtual world service. In particular, this paper investigated the representations of people with an impairment on external body parts. First, we analyzed the content of the interviews, followed by core-periphery analysis to identify the representations structure of the extracted topics (Borgatti and Everett, 2000). Finally, we mapped the topics in a maximum tree to clarify the relationship between the extracted topics (Flament, 1986).

### In-depth interviews

This study explored opinions of PWD on the embodiment of physical disorders with their avatars to answer a set of research questions. To collect data for analysis, we selected purposive sampling to recruit PWD with experience using the virtual world. This paper focused on Korean users to erase the cultural difference between social groups. Furthermore, Korea has a considerable interest in the virtual world market, while the level of disability awareness is relatively low (Kwon, 2021). According to the 2021 national human rights survey (National Human Rights Commission of Korea, 2021), 32.9% of the respondents chose PWD as the group that suffers the most human rights violations or discrimination, ranking second among 13 socially marginalized groups. As such, Korea is a representative example of the imbalance between technology’s rapid growth and society’s awareness of PWD. It is likely that Korean PWD have exposure to the virtual world, which is still in its early stages, and at the same time, they may have a perception of what risks the virtual world poses to PWD. Therefore, this study limited the scope of interviewees to Koreans.

Since this study’s main purpose is to examine the perception of how to implement avatars depicting disability in the virtual world, we recruited people with disabilities such as YouTubers because we estimated they had a high understanding of their disabilities. We made contact through e-mail or direct messages on Instagram from December 2021 to April 2022. However, the number of influencers who revealed their disabilities was very small. We made 38 contacts, of which nine responded affirmatively. While conducting interviews with nine respondents, we asked them to recommend someone who could participate in the interview among acquaintances with disabilities, and they introduced four more interviewees. Finally, we conducted in-depth interviews with 13 people with physical disabilities. The number of interviewees seems small. However, previous social representations theory-based studies could present meaningful results with similar size samples (Lai et al., 2017; Jang et al., 2021). Furthermore, paying attention to their opinions early in the virtual world market is necessary because avatars have a discriminatory meaning for PWD from general users (Davis and Chansiri, 2018).

All interviewees were in their 20s and 30s, and six were male. Ten interviewees had difficulty moving their legs, and the other three were hard of hearing. Since their external body parts had problems functioning, they used aids such as a wheelchair or cochlear implants, which were recognizable. Furthermore, all interviewees had experience using recent virtual world services accessible in Korea, such as ZEPETO, Gather.town, or ifland at least once. Table 1 shows detailed profiles of each interviewee.

**Table 1:** Interviewee information.

	Number of interviewees		Number of interviewees
Age		Virtual world service using experience	
20 s	8	One-time use	10
30 s	5	Once a week	1
Gender		Several times a week	1
Male	6	Almost daily	1
Female	7	Experienced service (allow multiple answers)	
Type of disability		ZEPETO	8
Hearing disability	3	Gather.town	2
Locomotor disability	10	Ifland	3
		Etc.	Roblox (1); Animal crossing (1)

We referred to Davis and Chansiri (2018) and prepared questions in advance on how PWD construct virtual identities with their avatars. Specifically, questions included the respondents’ willingness to express disorders with an avatar, reasons for the disability display or abandonment, preferred descriptions of disability, and so on. The interview was a semi-structured in-depth interview, so we continued with follow-up questions based on the interviewee’s answers. We delivered detailed explanations about the study and questionnaire in advance through e-mail. Interviewees were informed that they could withdraw their consent to participate whenever they wanted; in that case, their data would be excluded from the analysis. In addition, we provided interviewees a choice between offline and online interviews, considering the COVID-19 situation and several interviewees’ inconvenience of movement; only one interviewee chose the offline interview. Each interview lasted about 1 hour, and each interviewee received about 10 USD compensation (see Appendix A for detailed interview questions).

### Content analysis

We conducted a detailed coding of interviews for the analysis based on the process suggested by Jung and Pawlowski (2014). The first coder extracted topics from interview transcripts using an open coding process, extracting 29 codes. Next, the second coder independently coded the data based on the codes developed by the first coder. After this process, two coders reconciled disagreements on codes by combining codes with overlapping meanings or clarifying the meaning of each code. We used Cohen’s Kappa to check interrater reliability, achieving a value of 0.77, verifying that two coders had a consensus on the extracted topics (Altman, 1991). Finally, 16 topics were confirmed. Table 2 explains the final 16 topics related to avatar-based disability representations identified from the content analysis.

**Table 2:** Topics related to avatar-based disability representations.

No.	Topic	Example
T01	Display of physical flaws	• I want to create an avatar that embodies my physical disability. (Interviewee 5)
T02	Abandonment of physical flaws	• I do not want to make an avatar with a disability. (Interviewee 13)
T03	Freedom of choice	• I can customize the avatar in the way I want. (Interviewee 2) • It’s fun to be able to decorate my avatar freely. (Interviewee 9)
T04	Need to display	• Expressing disability is a matter of necessity rather than preference. (Interviewee 4) • Disabled avatars should exist in the virtual world. (Interviewee 1)
T05	Regarding as the fashion	• I hope the virtual world can recognize disability aids such as a wheelchair as a fashion item. (Interviewee 1)
T06	Improving disability awareness	• Non-disabled people will be able to become more familiar with disability by encountering them as avatars. (Interviewee 12)
T07	Support for real-world experience	• I want to show the lives of people with disability realistically. (Interviewee 3) • It would be good to rehearse physical activities in advance with avatars. (Interviewee 4)
T08	Smooth communication	• Since I have a hearing impairment, revealing a disability can have advantages in communication. (Interviewee 11) • Non-disabled people and disabled people will be able to interact naturally. (Interviewee 4)
T09	Strengthening prejudices	• If one misrepresents a disability, the non-disabled can misunderstand the disability. (Interviewee 1)
T10	Avoiding experienced risks	• I do not want to continue difficulties caused by the disability in reality in the virtual world. (Interviewee 9) • There is no need to deal with social stigma, even in the virtual world. (Interviewee 2)
T11	Enjoying the dreamed life	• I want to do something that I cannot do in reality, like running. (Interviewee 10)
T12	Beyond appearance	• Not only the appearance of the avatar but also the environment of the virtual world should be barrier-free. (Interviewee 6) • I hope the virtual world can show actions like sign language. (Interviewee 12)
T13	Fantasy description	• It would be fun to express disabilities like Iron Man. (Interviewee 6) • It would be nice to give a special ability to disability accessories. (Interviewee 8)
T14	Realistic description	• I hope the disability is implemented realistically (Interviewee 5)
T15	Offering a variety of options	• In addition to physical disorders, we must be able to express various disabilities. (Interviewee 12) • I wish I had a variety of options to represent my disability. (Interviewee 1)
T16	Blended into people	• I want other people to look at my avatar as a normal person. (Interviewee 7)

### Analysis of the structure: core and periphery analysis

The next step of the analysis was clarifying the structure of 16 topics for avatar-based disability representations. It is crucial to identify the structure of social representations based on core and peripheral elements (Abric, 1993). The central core is a stable and preserved part of the representations, while the peripheral indicates a changeable and individual-related part of representations (Abric, 2001). Following prior studies on a core-periphery structure of social representations (Jang et al., 2021), we adopted the core-periphery algorithm developed by Borgatti and Everett (2000) to discover a core-periphery structure from network data. We used the statistical software UCINET for analysis. Next, we divided topics into core and periphery groups based on each element’s coreness, indicating each element’s extent of association with the latent center (Borgatti and Everett, 2000). Finally, we used a co-occurrence matrix for analysis.

### Mapping social representations

We visualized the structure of the social representations based on the former analysis and formed a maximum tree by connecting two elements based on the similarity coefficient (Flament, 1986). First, we selected Jaccard’s similarity coefficient, which operationalized the degree of agreement based on co-occurrence between elements (Hammond, 1993). We then started mapping by finding an element X with the largest frequency value. Next, we linked X to another element with the highest similarity coefficient with X. Finally, we repeated these steps until we connected all 16 topics.

## Results and discussion

**Figure 2:**
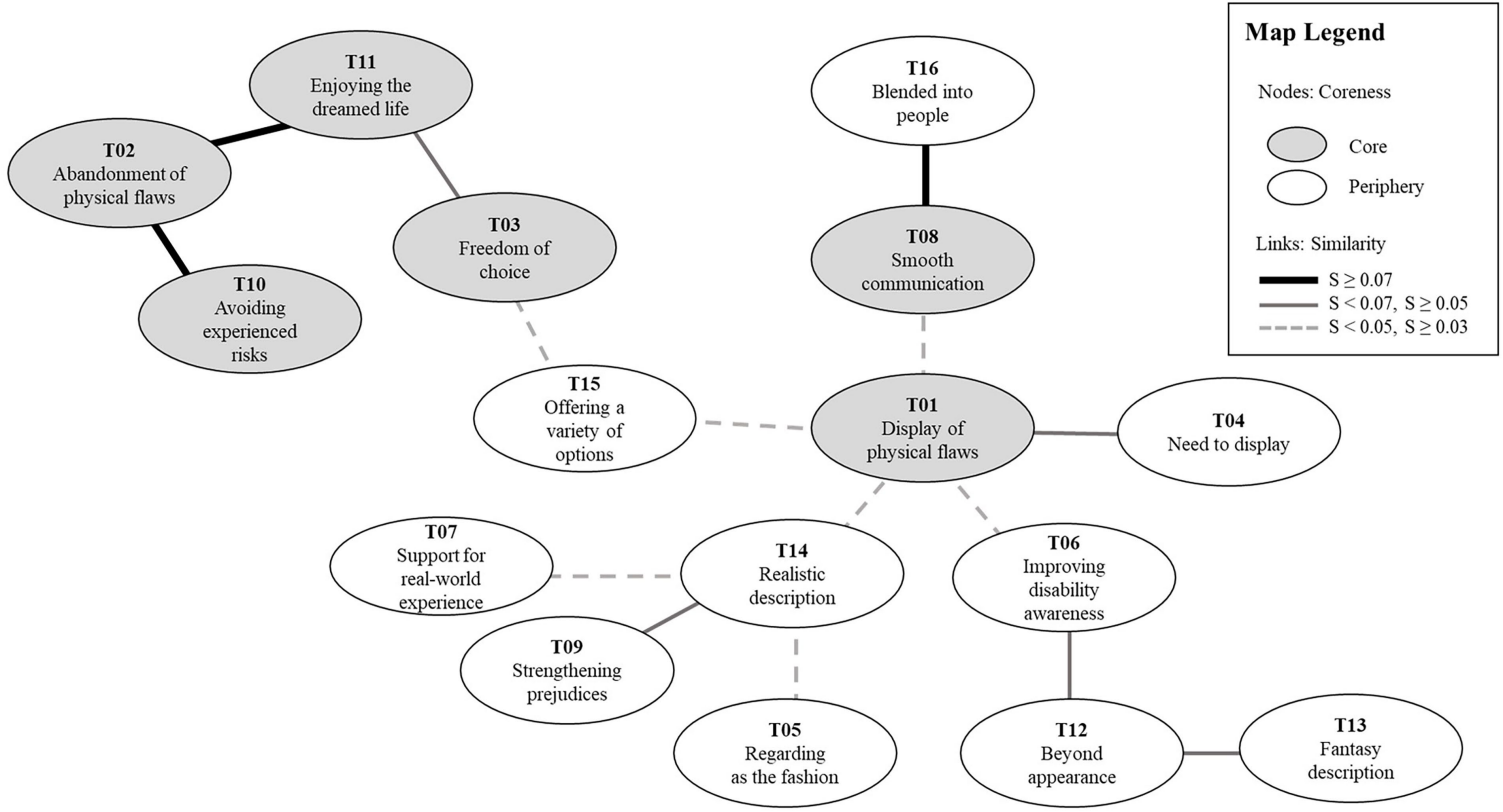
Social representations map.

The content analysis results identify 16 topics related to avatar-based disability representations considered by PWD. Based on the coreness value of each topic measured by the core-periphery analysis, we defined six topics as core and the other ten as periphery. Table 3 shows the coreness value of each topic. The core topics are as follows: abandonment of physical flaws (T02), enjoying the dreamed life (T11), avoiding experienced risks (T10), smooth communication (T08), freedom of choice (T03), and display of physical flaws (T01). The social representations in Figure 2 show a comprehensive perceptual map of PWD about avatar-based disability representations in the virtual world.

**Table 3:** Core-periphery structure.

No.	Topic	Coreness	Membership
T02	Abandonment of physical flaws	0.586	CORE
T11	Enjoying the dreamed life	0.581	
T10	Avoiding experienced risks	0.300	
T08	Smooth communication	0.270	
T03	Freedom of choice	0.218	
T01	Display of physical flaws	0.202	
T12	Beyond appearance	0.116	PERIPHERY
T09	Strengthening prejudices	0.116	
T06	Improving disability awareness	0.106	
T05	Regarding as the fashion	0.103	
T04	Need to display	0.094	
T13	Fantasy description	0.082	
T15	Offering a variety of options	0.053	
T14	Realistic description	0.035	
T07	Support for real-world experience	0.010	
T16	Blended into people	0.004	

We provided a comprehensive and detailed perception of PWD on avatar-based disability representations as the result of the social representations approach. In this section, we demonstrated a willingness to express disorders with an avatar, reasons for the disability display or abandonment, and preferred descriptions of disability from the perceptual map to answer the research questions. We adopted Davis and Chansiri’s (2018) three paradigms for virtual identity construction for discussion. We highlighted abandonment of disability corresponding to paradigm one and display of disability corresponding to paradigm three on the map. We confirmed the second paradigm, indicating selective expression of physical flaws depending on the situation, by investigating topics connecting the first and third paradigms on the map. In addition, we examined preferred ways and reasons for abandonment or display of physical flaws.

### Abandonment of physical flaws

Abandonment of physical flaws (T02) had the highest coreness score among the 16 topics and shared high similarities with core topics. This high coreness value answered RQ_1_ regarding a willingness to display physical flaws; the negative perspective on disclosing disability through avatars appeared to be more central to PWD. There was a close link between abandonment of physical flaws (T02) with avoiding experienced risks (T10) and enjoying the dreamed life (T11). These relationships between topics could explain RQ_2_ about reasons for disability abandonment in this context. PWD face a lot of barriers, including physical impediments to social stigma in the actual world, which restricts them from various activities. They try to avoid the risk of encountering obstacles that they experienced in the real world by hiding their disability in the virtual world. However, they believed that disability barriers would still exist in the virtual world and did not want to challenge them.

**Figure 3:**
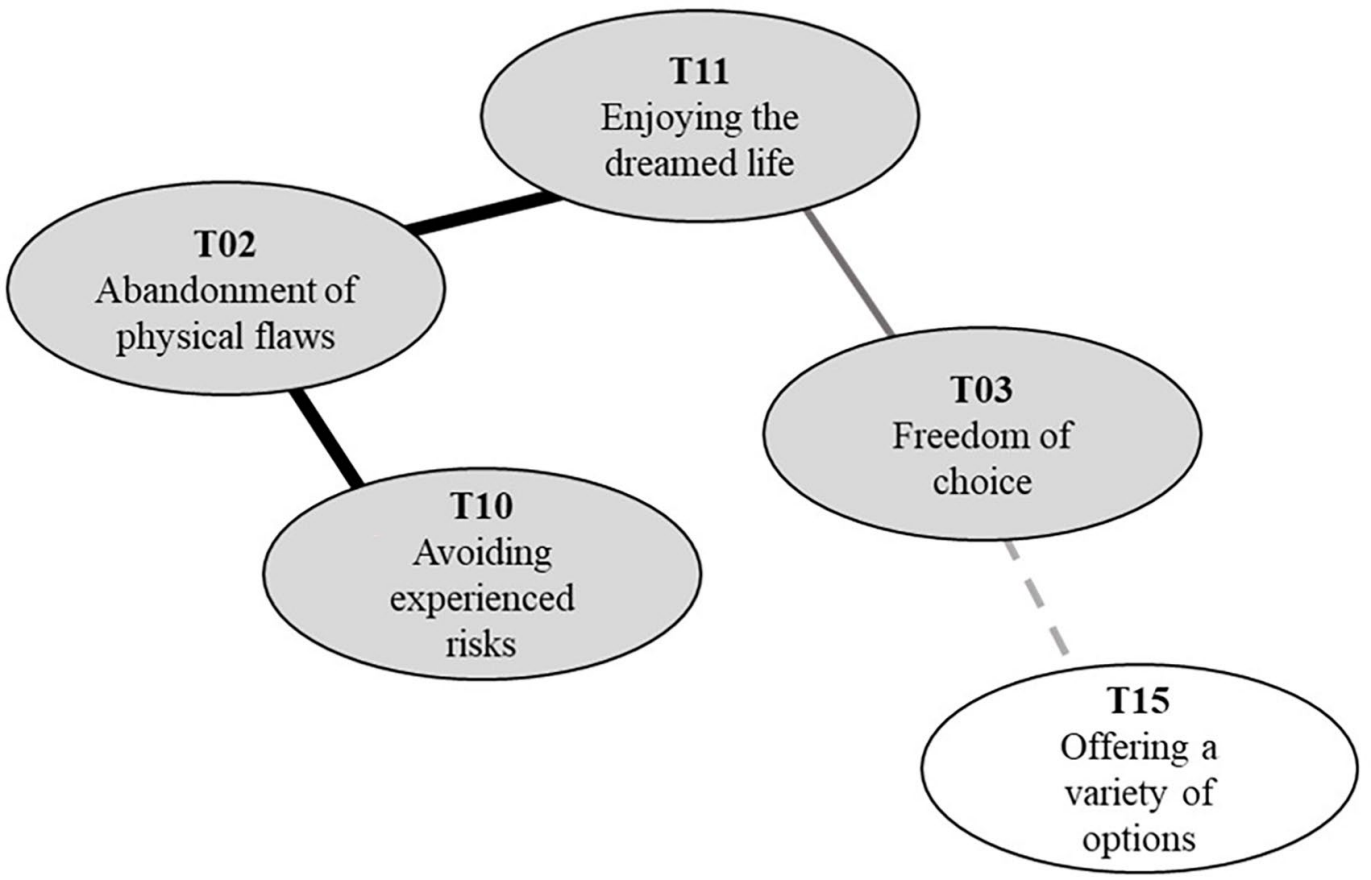
Negative perspective on avatar-based disability representations.

There was also a strong connection between enjoying the dreamed life (T11) and abandonment of physical flaws (T02). PWD put avatars as digital substitutes to do what they cannot do in real life. For example, they mentioned they wanted to go to places with many stairs or enjoy sports such as running or swimming. Therefore, to enjoy the virtual world of their dreams, they needed differently-constructed bodies than in reality. PWD emphasize the freedom of choice (T03) as the key trait of avatar customization that enables enjoying the dreamed life (T11). For the PWD, born with undesirable physical traits, freedom of choice for their virtual body was the basis for a dream life. It allows one to consider preferences in deciding what to do instead of possibility. See Figure 3 for a part of the map explaining the negative perspective on avatar-based disability representations.

### Display of physical flaws

**Figure 4:**
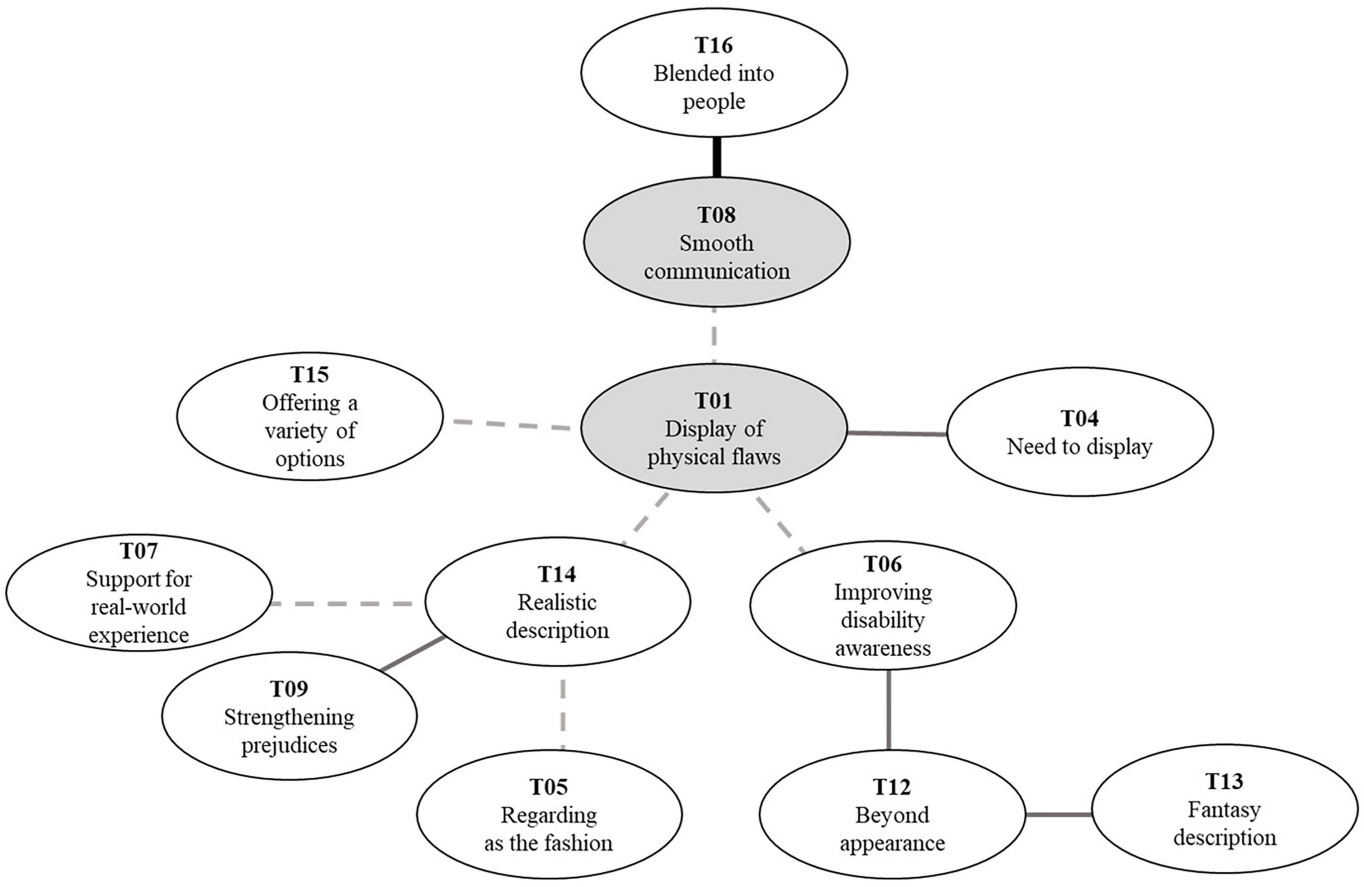
Positive perspective on avatar-based disability representations.

Despite a willingness to conceal disability taking the core of the perceptual map, PWD perceive the positive aspects of displaying physical flaws. This part of the map supplemented the answer to RQ_1_ by showing the positive perspective on disability embodiment (Figure 4).

We classified the display of physical flaws (T01) as a core element with the highest similarity value. It meant that PWD cared about reflecting disability in virtual identity construction even though it was not in the middle of their perception. Display of physical flaws (T01) did not closely link to specific topics, but PWD considered it along with various topics. We discovered diverse reasons and preferred ways to disclose their physical flaws from these links as the answers to RQ_2_ and RQ_3_. First, we found a link between the display of physical flaws (T01) and the need to display (T04) and smooth communication (T08). Need to display (T04) differed from freedom of choice (T03) in explaining the disability embodiment as an area of need, not an area of preference.

Several interviewees claimed it is necessary to reveal disabilities and said they feel obligated and responsible for revealing their disabilities in the virtual world. For PWD with hearing impairment, it was also necessary to display physical flaws for smooth communication (T08). Deafness turned into difficulty when PWD communicated with others. PWD with hearing impairment felt they should mention that they have difficulties in voice-based communication during interactions with others. By wearing cochlear implant identifiers on their avatar, the PWD with hearing impairment could naturally express their disability without mentioning it. Other people may perceive that they need to chat in text instead of voice with avatars wearing cochlear implant identifiers, leading to smooth communication. However, smooth communication (T08) is closely related to the desire to blend with people (T16). PWD wanted to blend with people (T16) when communicating with others even though they revealed their physical impairments. In other words, the PWD wanted others to notice their disabilities to the extent of functional purpose for facilitating communication.

Display of physical flaws (T01) is also related to the understanding of disability positively and negatively. On the positive side, disability representations link directly to improving disability awareness (T06). The non-disabled could be more familiar with the disability by being exposed to avatars with disability in the virtual world. In particular, given that the virtual world services were popular among young users, the young generation could be exposed to disability from an early age. Respondents frequently mentioned improving disability awareness (T06) with beyond appearance (T12). This linkage explained the need for implementing disability awareness in the virtual world environment or the movement of avatars beyond the external appearance of avatars. Schultze (2014) claimed that identity might not exist in one’s physical body but in the people they communicate with and the place they reside.

Therefore, to help PWD establish their virtual identity related to disability in the way they want, the virtual world’s environment should reflect consideration and provide disability identifiers. In describing their appearance and traits beyond appearance (T12), they preferred fantasy description (T13), providing disability identifiers in a novel way that does not exist in reality (e.g., ZEPETO). They expected a new shape to describe the disability with a new ability. For example, describing disorders with moving legs in the form of robot legs adds the ability to fly if avatars wear a corresponding identifier. In this case, one can view disability as a characteristic, not a difficulty, unlike reality. It will also open the opportunity for non-disabled people to choose an identifier that expresses disability.

On the other hand, there were concerns about strengthening prejudices (T09) derived from the display of physical flaws in a realistic way (T14). Realistic description (T14) involved identifiers resembling the appearance of disability aids (e.g., Gather.town). There were concerns that if PWD describe disabilities realistically, social bias on disabilities will extend to the virtual world. Several interviewees worried that if the virtual world implemented physical impairment realistically, others would treat the avatar as a passive being needing help even though their physical traits do not limit their actions in the virtual world. Accordingly, they hoped one would regard realistically-implemented disability aids as fashion items (T05). PWD try to express their individuality through disability aids identifiers by selecting color or designs, while the form of disability aids follows the realistic shape.

Realistic description (T14) of disability also has a functional purpose. PWD prefer to illustrate their physical impairments similar to reality to support real-world experience (T07). Some interviewees mentioned that in enjoying the virtual world as a space preceding real-world experiences, one needs to implement the physical body in the virtual world as they are in the real world. For example, PWD could rehearse the activity in the virtual world before doing it in reality to reduce the danger in the physical world. To achieve this goal, the PWD should embody their disability. See Figure 4 for a part of the map explaining the positive perspective on avatar-based disability representations.

### The bridge between two perspectives

**Figure 5:**
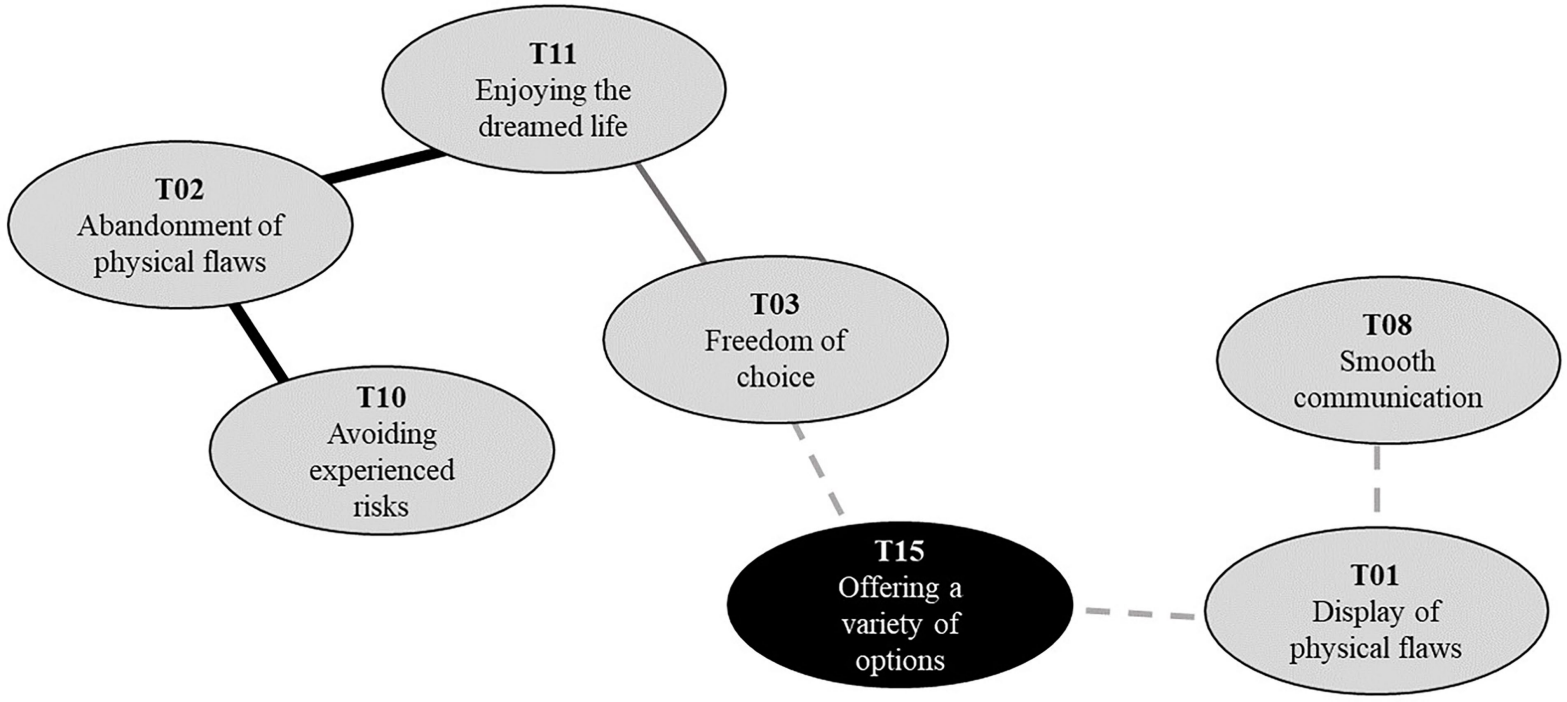
Bridge topic (colored black) connecting incompatible perspectives on avatar-based disability representations.

We cannot place at once abandonment of physical flaws (T02) and display of physical flaws (T01). We formulated two distinct concepts based on different sets of topics; however, a topic offering various options (T15) played the role of a bridge. Regardless of whether PWD wanted to reveal their disabilities, all the PWD agreed that the virtual world should provide diverse disability identifiers as options. Currently, there are limited options for PWD to implement their disorders. Offering different options (T15), where two perspectives come across, represented the implicit consensus among persons with disabilities that there should be various alternatives for expressing their disorders regardless of whether they define or identify them. Individual users select given resources for avatar customization to express themselves in the way they want (Sundar and Marathe, 2010). Therefore, sufficient identifiers were the basic resources required for implementing physical flaws. See Figure 5 for a black-colored bridge topic that connected the two incompatible perspectives.

## Conclusion

This study contributes to a better understanding of avatar-based disability representations from disabled users’ perspectives by employing the SRT, which identifies shared awareness of emerging social objects between particular social groups. Among several options for SRT, we selected the core-periphery approach employed in previous studies to identify social representations successfully (Ahn and Jung, 2016; Jang et al., 2021). This study identified the social representations of people with external physical disabilities on the social object, the avatar, based on the appropriate theory and systematic approach. The study’s comprehensive results could answer the main research question—how do people with physical disabilities want to construct virtual identities with avatars? The PWD’s perception of avatars consists of two conflicting opinions on displaying physical flaws in avatars.

First, the core-periphery analysis identifies the negative perspective on disclosing physical impairments as the core of the PWD shared perception of avatars. They tend to hide their physical flaws in the virtual world, where they fully control their body. Even though their disorder might not restrict their activity in the physical world, they worry that reproducing their disabilities in the virtual world through disabled avatars would create barriers as in the physical world. Based on favorable avatars, they want to enjoy the life they desire, corresponding to previous findings that the PWD felt satisfied and pleased with their life by creating an ideal avatar without disability (Hu et al., 2015).

Second, there is a positive link relating to the display. Positively, PWD anticipate that the young generation interacting with disabled avatars in the virtual world will be familiar with disability. They will accept disability naturally instead of establishing prejudices on disability based on difficulties they notice in the physical world. Additionally, they suggest expanding the range of disability representations to the movement or environment of the virtual world. Disability goes beyond the physical discomfort PWD experience. Disability lies in the social interactions between individuals and their environment (Schultze, 2014). For example, hearing impairment does not cause many difficulties to the individual unless they communicate with others. Therefore, to properly implement the disability, it is necessary to reflect disability awareness in various contexts beyond revealing the disability through an avatar’s appearance.

Third, the degree of reality for describing the disability changes based on the context. For example, PWD describe physical impairments realistically when they want to practice real-world experience in the virtual world. However, they also worry about social bias expanding to the disorder in the virtual world by illustrating disabilities similar to the real world. Conversely, PWD suggest a fantasy description to describe disability comprehensively rather than just illustrating the appearance. In addition, they expect the virtual world to represent the disability as a special ability rather than a difficulty, thus closely linking the representation with improving disability awareness.

Lastly, whether they are willing to reveal their physical traits, PWD have a shared understanding of the necessity of diverse alternatives to embody disability. They had conflicting opinions about revealing disabilities but agreed on the need for various options for displaying disabilities. Sundar and Marathe (2010) described avatar customization as expressing identity in a satisfying format with provided resources. Individuals only can freely establish their virtual identity when the resources offer sufficient identifiers. Hence, apart from their preferences, the PWD agreed that the virtual world should provide options for various disorders to express disability identity.

This study contributes to the academic studies of avatar-based representations of disability, filling the gap in the literature on virtual representations of PWD. It provides a shared understanding of avatars among users with physical disabilities. Furthermore, since prior studies only conducted interviews with users of Second Life, they likely had bias derived from the characteristics of the service. By showing disabled users’ social representations of the avatar-based disability representations, this study clarifies their current perspectives on virtual disability embodiment. In particular, our research answers whether the PWD want to display their physical flaws with avatars, why they want to display or abandon them, and their preference for disability description. The findings support the three paradigms of reconstruction, combination, and implementation of physical identity in virtual identity construction suggested by Davis and Chansiri (2018) in the context of disability representations.

This paper also has practical implications for building a better virtual world service. The core-periphery analysis results indicate that topics related to the abandonment of disability get a high coreness value. The research result implies that PWD hesitate to disclose their disabilities in a virtual world because of worries about facing the same risks they experience in the physical world. They do not regard the current virtual world as a safe place where the appearance of their avatars discriminates against no one. For instance, Lee and Park (2011) worried about the detachment of socially marginalized groups from the virtual world since they could not find other avatars that shared their physical attributes. As a result, they felt isolated even in cyberspace. We can extend the negative effect of numerical underrepresentation of social minorities (Lee and Park, 2011) to the idea that increasing the numerical number of avatars disclosing physical weakness might be the starting point of a solution.

The social representations map shows that a topic links paradigms one and three, offering different options. Virtual world service providers should lead the PWD to try to embody their disability with avatars by providing a variety of disability identifiers. Building an environment where the PWD can reveal their disabilities freely is also important from the perspective of users without disabilities. Unlike constructing appropriate avatars for mission accomplishment in games, users have the freedom to create their avatars while using social virtual world services without special missions (Jung and Pawlowski, 2014). Users enjoy communicating with others as their main activity in this world, and cheating occurs during this process. For example, despite its ground-breaking concept, Second Life has suffered from various issues such as sexual harassment of child avatars or romantic relationships between avatars that conflicted with the real-world couple. Since the virtual world reflects reality, the gap between a life based on the physical body and a life based on the virtual body causes problems. Similar problems can occur in the case of people with disabilities.

Thus, PWD should freely reveal or hide their disability through their avatar. However, if users communicating with a non-disabled avatar find out that the avatar’s user looks completely different from the avatar, they might doubt the trustworthiness of this virtual world. One could treat the virtual world, which does not mirror the physical world, as a space of cheating where one could not properly implement anything in reality. However, there is a lack of understanding of representations of physical constraints in the virtual world. People not isolated with their physical traits would not have critical difficulties constructing avatars that reflect their real-world appearance. The problem is those constrained by their physical traits. Therefore, it is necessary to pay more attention to social minorities’ opinions on reducing their risk of displaying their physical traits to make the virtual world more authentic. The PWD’s shared perception of avatar-based disability representations could be a good starting point. Accordingly, considering the finding of this study, the virtual world service should respond to the more fundamental question about the authenticity of the virtual world by paying attention to PWD.

This study has some limitations requiring further research. First, a small number of interviewees were involved in the analysis. Researchers should conduct quantitative research based on the survey as the virtual world becomes popular and the number of users increases. Second, this study only dealt with the case of South Korea. Even though South Korea is highly interested in the virtual world, understanding the avatar-based representations of disability may differ in other countries due to differences in social understandings of disabilities. Finally, future studies should compare how users with different origins react to the disclosure of disability in the virtual world.

## Data availability statement

The original contributions presented in the study are included in the article, further inquiries can be directed to the corresponding author.

## Ethics statement

Ethical review and approval was not required for the study on human participants in accordance with the local legislation and institutional requirements. The patients/participants provided their written informed consent to participate in this study.

## Author contributions

JP suggested the topic for the article and wrote the article. SK suggested the theoretical background for the article and reviewed the article. JP and SK conducted data analysis and interpretation with discussion. All authors contributed to the article and approved the submitted version.

## Funding

This study was supported by the Ministry of Education of the Republic of Korea and the National Research Foundation of Korea (NRF-2019S1A3A2099973) and the MSIT (Ministry of Science and ICT), Korea, under the ITRC (Information Technology Research Center) support program (IITP-2020-0-01749) supervised by the IITP (Institute of Information & Communications Technology Planning & Evaluation).

## Conflict of interest

The authors declare that the research was conducted in the absence of any commercial or financial relationships that could be construed as a potential conflict of interest.

## Publisher’s note

All claims expressed in this article are solely those of the authors and do not necessarily represent those of their affiliated organizations, or those of the publisher, the editors and the reviewers. Any product that may be evaluated in this article, or claim that may be made by its manufacturer, is not guaranteed or endorsed by the publisher.

## Appendix

### APPENDIX A Questions for interview.

**Table:**

Questions for disability
• What kinds of physical disabilities do you have? • Is it a congenital disorder or an acquired disorder? • What kinds of difficulties do you experience in real life?
Questions for the virtual world usage
• Which virtual world service have you used? • How often have you used virtual world service? • What was the purpose of using virtual world service?
Questions for avatar-based disability representations
• What keywords come to mind when you think of the word ‘avatar?’ • What keywords come to mind when you think of the word ‘avatar-based disability representations’? • Do you want to disclose your disability with avatars in the virtual world? • If you want/do not want to disclose your disability with avatars in the virtual world, what is the reason for it? • If you want to disclose your disability with avatars, how do you want to display it?
